# The Prader-Willi syndrome murine imprinting center is not involved in the spatio-temporal transcriptional regulation of the Necdin gene

**DOI:** 10.1186/1471-2156-6-1

**Published:** 2005-01-05

**Authors:** Françoise Watrin, Elodie Le Meur, Nathalie Roeckel, Marie-Anne Ripoche, Luisa Dandolo, Françoise Muscatelli

**Affiliations:** 1 Centre National de la Recherche Scientifique UMR 6156, IBDM, Parc scientifique de Luminy, Case 907, 13288 Marseille Cedex 9, France; 2 Institut National de la Santé et de la Recherche Médicale UMR491, IPHM, Faculté de Médecine de la Timone, 27 Bd. J. Moulin, 13385 Marseille Cedex 5, France; 3 Département de Développement, Génétique et Pathologie Moléculaire, Institut Cochin, 24 rue du Faubourg St Jacques, 75014 Paris, France

## Abstract

**Background:**

The human Prader-Willi syndrome (PWS) domain and its mouse orthologue include a cluster of paternally expressed genes which imprinted expression is co-ordinately regulated by an imprinting center (IC) closely associated to the *Snurf*-*Snrpn *gene. Besides their co-regulated imprinted expression, two observations suggest that the spatio-temporal expression of these genes could also be co-regulated. First, the PWS genes have all been reported to be expressed in the mouse nervous system. Second, *Snurf*-*Snrpn *and its associated IC are the most ancient elements of the domain which later acquired additional functional genes by retrotransposition. Although located at least 1.5 megabases from the IC, these retroposons acquired the same imprinted regulation as *Snurf*-*Snrpn*. In this study, we ask whether the IC, in addition to its function in imprinting, could also be involved in the spatio-temporal regulation of genes in the PWS domain.

**Results:**

We compared the expression pattern of *Snurf*-*Snrpn *and C/D-box small nucleolar RNAs (snoRNAs) *MBII*-*85 *and *MBII*-*52 *to the expression pattern of the two evolutionary related retroposons *Ndn *and *Magel2*, in the developing mouse embryo. We show that these genes have highly similar expression patterns in the central nervous system, suggesting that they share a common central nervous system-specific regulatory element. Among these genes, *Ndn *and *Magel2 *display the most similar expression patterns. Using transgenic mice containing the *Ndn *and *Magel2 *genes, we show that the transgenic *Ndn *gene whereas not imprinted is correctly expressed. Search for DNase I hypersensitive sites in the *Ndn*-*Magel2 *genomic region and comparative genomic analyses were performed in order to identify potential transcriptional *cis*-regulatory elements.

**Conclusions:**

These results strongly suggest that paternally expressed genes of the PWS domain share a common central nervous system-specific regulatory element. We proposed that this regulatory element could co-localize with the IC. However, we demonstrate that the IC, if required for imprinted regulation, is not involved in the spatio-temporal regulation of distantly located retrotransposed genes such as the *Ndn *gene in the PWS domain.

## Background

Genomic imprinting in mammals is a process that leads to the preferential mono-allelic expression of specific genes in diploid cells, depending on whether they are inherited from the sperm or from the oocyte. To date, approximately 70 mammalian imprinted genes have been identified which map to at least 11 regions of the mouse genome [1]. Most imprinted genes are therefore located in clusters, which are generally conserved between human and mouse. For some of these clusters, coordinate imprinted gene regulation has been shown to be controlled by imprinting centers (IC) [2]. Imprinted genomic regions from the two parents are differentially marked by heritable epigenetic modifications including DNA methylation and histone acetylation and/or methylation. These epigenetic modifications or imprints are established at least for some of them in the germ line of either parent [2].

The Prader-Willi syndrome (PWS) domain on human chromosome 15q11-q13 and its ortholog on mouse chromosome 7C-D1 are large chromosomal domains containing paternally expressed genes [3]. PWS results from the loss of expression of several of them including *SNURF*-*SNRPN*, *NDN*, *MAGEL2*, *MKRN3 *and the C/D-box small nucleolar RNAs(snoRNAs). In humans, mini-deletions, upstream the *SNURF*-*SNRPN *transcriptional unit, have led to the characterization of an IC which coordinates their imprinted expression [4]. One sub-region of this IC defined as the shortest region of deletion overlap in PWS patients (PWS-SRO) and encompassing the *SNURF*-*SNRPN *promoter, has been shown to be required for maintaining the paternal imprint [5,6]. Its deletion in the germ line or post-zygotically on the paternal chromosome leads to silencing of all paternally expressed genes of the PWS domain [4,6]. In mice, although the *Snrpn *promoter is not required for genomic imprinting [7], deletion of a 35 kb region including 16 kb of sequences upstream *Snurf*-*Snrpn *exon 1 to *Snurf*-*Snrpn *exon 7 unit also leads to silencing of the paternally expressed genes, indicating that both the position of the IC and its role in the coordinate imprinted expression of genes is conserved between human and mouse [8,9].

Recently, examination of tissue-specific pattern of mRNA expression at a genomic scale allowed the identification of several chromosomal regions harbouring tissue-specific co-regulated genes defined as regions of correlated transcription (RCTs) [10]. Noticeable, some of these RCTs overlap with known imprinted loci and it suggests that transcriptional regulatory elements controlling imprinted expression might also regulate tissue-specific expression. Therefore, one might hypothesize that this type of regulation is applied at the PWS domain. Two arguments support this hypothesis. First, all the murine orthologues of the PWS genes have been reported to be expressed in the developing nervous system [11]. Second, Nicholls proposed an evolutionary model for the origin of the human and mouse PWS domains [12,13]. In this model, the *Snurf*-*Snrpn *locus and its associated IC would be the ancestral imprinted transcriptional unit of the domain. The other genes, which are intronless, would have later and sequentially been acquired by retrotransposition and adopted the same imprinted regulation. Altogether, these data suggest that a spatio-temporal co-regulation of PWS genes could exist and have been acquired through the evolution of the domain. Thus, it is tempting to speculate that the *cis*-regulatory element(s) initially involved in the spatio-temporal regulation of the *Snurf*-*Snrpn *locus might influence the spatio-temporal regulation of retrotransposed genes in the PWS region. The IC could be a good candidate to play this role.

In this study, we investigate this hypothesis. First, we compared the expression pattern of the *Snurf*-*Snrpn*, *MBII*-*85*, *MBII*-*52*, *Ndn *and *Magel2 *in the developing mouse embryo. We show that their expression in the embryo is restricted to neural tissues and that these genes display strikingly similar expression patterns in the central nervous system. Second, we created transgenic mice with a BAC containing the *Ndn *and *Magel2 *genes. We showed that the *Ndn *transgene is not imprinted but is correctly expressed in the developing embryo. These results demonstrate that if the IC is required for imprinted regulation, it is not involved in the *Ndn *spatio-temporal regulation. Finally, since we have shown an almost identical spatio-temporal expression profile of *Ndn *and *Magel*2 in the developing embryo, it suggests that these two genes might share common *cis*-acting regulatory elements which should be present in the BAC transgene. We therefore searched for DNaseI hypersensitive sites in the *Ndn*-*Magel2 *genomic region and performed comparative genomic analyses in order to identify potential transcriptional *cis*-regulatory elements.

## Results

### Nervous system tissue-specific expression of mouse PWS genes in the embryo

**Figure 1:**
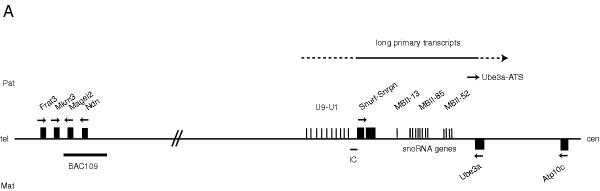
**Imprinted cluster on mouse chromosome 7C. **Upper and lower boxes represent genes expressed from the paternal and maternal alleles respectively. Small nucleolar RNAs (snoRNAs) *MBII*-*13*, *MBII*-*85 *and *MBII*-*52 *genes lie within introns of long primary transcripts initiated at the U exons (U9 to U1 exons). Arrows indicate the transcriptional orientation of the genes. *Frat3*, *Mkrn3*, *Magel2 *and *Ndn *lie within 120 kb and around 1.5 to 2 Mb from the *Snurf*-*Snrpn *gene. Location of BAC109 used for the transgenic study is represented.

**Figure 2:**
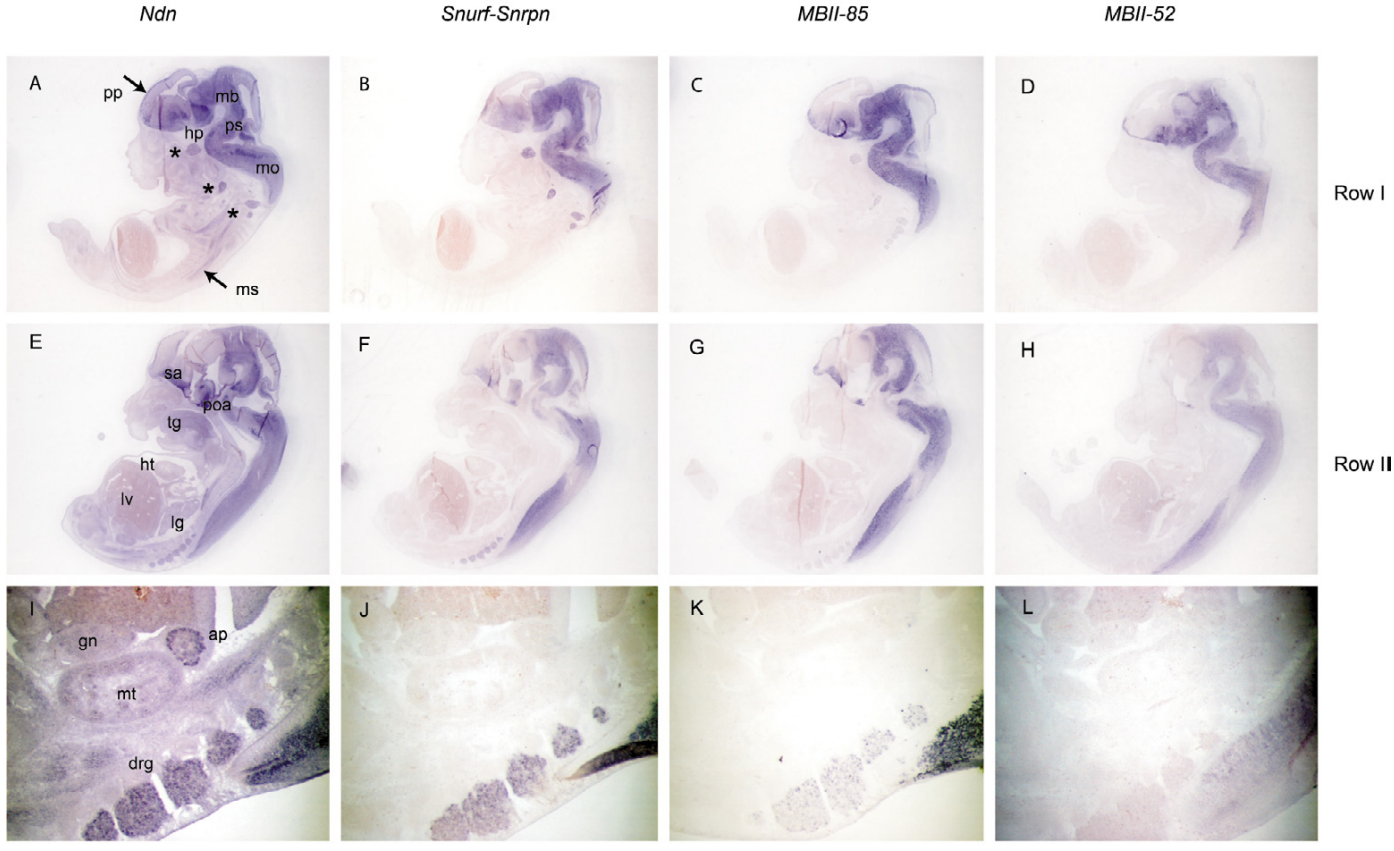
**Comparison of *Ndn* and *Snurf-Snrpn* transcriptional unit expression patterns in the E13.5 mouse embryo. **Comparison of *Ndn *(A, E, I), *Snurf*-*Snrpn *(B, F, J), *MBII*-*85 *(C, G, K) and *MBII*-*52 *(D, H, L) RNA expression on E13.5 mouse embryo adjacent sagittal sections, from lateral to more medial sections (rows I to II). *Ndn *as well as *Snurf*/*Snrpn*, *MBII*-*85 *and *MBII*-*52 *are almost exclusively expressed in the developing nervous system and display strikingly similar expression patterns in the developing brain and spinal chord. In the peripheral nervous system, *Ndn *and *Snurf*-*Snrpn *are expressed at similar levels in cranial (*Ndn*: A; *Snurf*-*Snrpn*: B) and dorsal root ganglia (*Ndn*: I; *Snurf*-*Snrpn*: J) whereas *MBII*-*85 *(C, K) and *MBII*-*52 *(D, L) are expressed at much lower levels or not at all. Note that *Ndn *is additionally expressed in muscle tissues such as the skeletal muscles (A) and the tongue (E), and in the adrenal primordium (I). ap, adrenal primordium; drg, dorsal root ganglia; gn, gonad; hp, hypothalamus; ht, heart; lg, lung; lv, liver; mb, midbrain; mo, medulla oblongata; mt, metanephros; ms, muscles; poa, post-optic area; pp, telencephalic epithelium preplate; ps, pons; sa, septal area; tg, tongue. Asterics indicate cranial and dorsal root ganglia in A, B, C, D.

**Figure 3:**
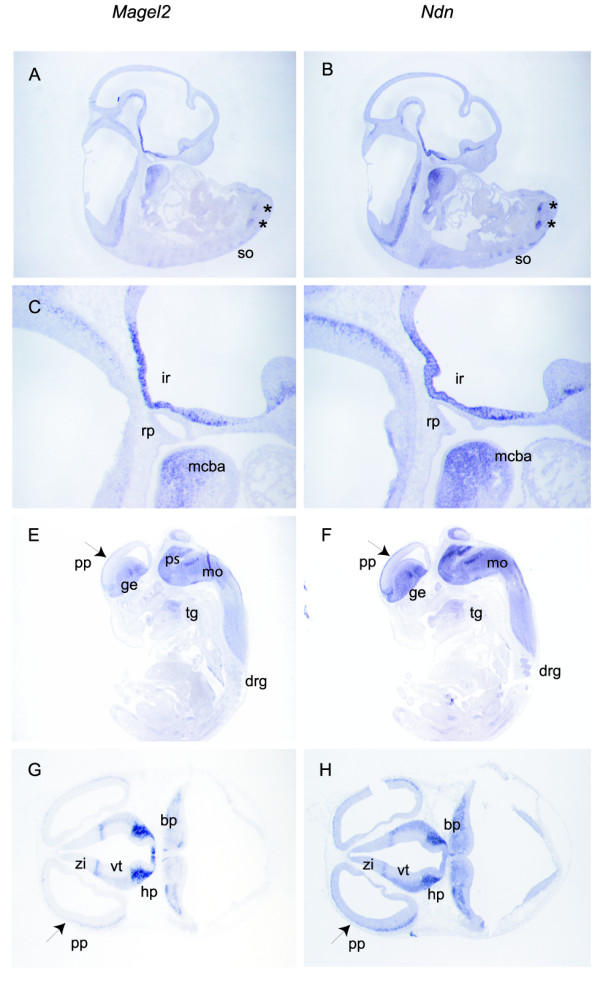
**Comparison of *Magel2* and *Ndn* expression patterns in the E10.5 and E12.5 mouse embryo. **Comparison of *Magel2 *(A, C, E, G) and *Ndn *(B, D, F, H) RNA expression in E10.5 (A, B, C, D) and E12.5 (E, F) mouse embryos on adjacent sagittal sections, and in E12.5 (G, H) mouse embryos on adjacent transversal sections. In the E10.5 embryo, the predominant expression in the ventral parts of the neural tube is particularly evident (A, B, C, D) for both genes. *Magel2 *and *Ndn *display a strikingly similar profile of expression in the infundibulum recess (C, D). Asterics indicate expression of both genes in symetrical ventral stripes of cells in the neural tube (A, B). Note that *Magel2 *as *Ndn *are expressed in the mandibular component of the first branchial arch (C, D), in the tongue (E, F). In the E12.5 embryo, *Magel2 *and *Ndn *are expressed in identical structures such as the ganglionic eminence, the pons, the medulla oblongata, in the preplate of the telencephalic vesicle, the hypothalamus, the ventral thalamus, the zona limitans intrathalamica and in the basal plate (E, F, G, H). drg, dorsal root ganglia; ge, ganglionic eminence; hp, hypothalamus; mcba, mandibular component of the first branchial arch; mo, medulla oblongata; pp, telencephalic epithelium pre-plate; ps, pons; rp, rathke pouc;h so, somites; tg, tongue; vt, ventral thalamus; zi, zona limitans intrathalamica.

In order to determine if mouse PWS genes might be transcriptionally co-regulated, we compared the expression profiles of *Snurf*-*Snrpn*, *MBII*-85, *MBII*-52, *Ndn *and *Magel2 *by *in situ *hybridization in the developing mouse embryo (Fig. 1, 2 and 3). In the 10.5 and 13.5 mouse embryos, *Snurf*/*Snrpn*, *MBII*-*85*, *MBII*-*52*, *Ndn *and *Magel2 *are almost exclusively expressed in the developing nervous system, and although expressed at different levels, they display strikingly similar expression patterns in the central nervous system (brain and spinal chord). As previously reported for *Ndn *[14], they are predominantly expressed in all the ventral parts of the neural tube, mostly or even exclusively in marginal areas where differentiating neurons reside. In the peripheral nervous system, *Ndn *and *Snurf*/*Snrpn *are both expressed at high levels in cranial and dorsal root ganglia, sympathetic and parasympathetic ganglia, structures in which *MBII*-*85*, *MBII*-*52 *and *Magel2 *trancripts are expressed at much lower levels or not at all (Fig. 2 and 3). *Magel2 *when compared to *Ndn*, *Snurf*-*Snrpn *and *MBII*-*85*/ -*52 *seems to have a more highly restricted expression domain. In the 12.5 embryo, *Magel2 *transcripts are detected at high levels and predominantly in the hypothalamus (Fig. 3). Careful examination shows that although found at low or very low levels, *Magel2 *transcripts are present in similar domains as the four other PWS genes studied. *Ndn *and *Magel2 *also share expression domains in some non-neuronal tissues such as in the muscles (skeletal muscles and tongue) and in some non-neuronal neural crest cells derived structures such as the branchial arches (Fig. 3; data not shown). *Magel2 *transcripts are detected at very low levels in the dorsal root ganglia and not detected in cranial ganglia.

In conclusion, all the PWS genes that we studied were expressed mainly (*Ndn *and *Magel2*) or even exclusively (*Snurf*-*Snrpn*, *MBII*-*85*/-*52*) in the mouse developing nervous system at E12.5 and E13.5, with strikingly similar identical patterns in the central nervous system (brain and spinal chord). More divergent expression patterns were observed in the peripheral nervous system (cranial and dorsal root ganglia, sympathetic ganglia), some genes being expressed at high levels (*Ndn *and *Snurf*-*Snrpn*) and the others being expressed at low levels or not at all (*MBII*-*85*, *MBII*-*52 *and *Magel2*). Our *in situ *hybridization data suggests the presence in the PWS domain of a central nervous system-specific neural element which coordinates the PWS genes expression.

### Transgenic experiments

**Figure 4:**
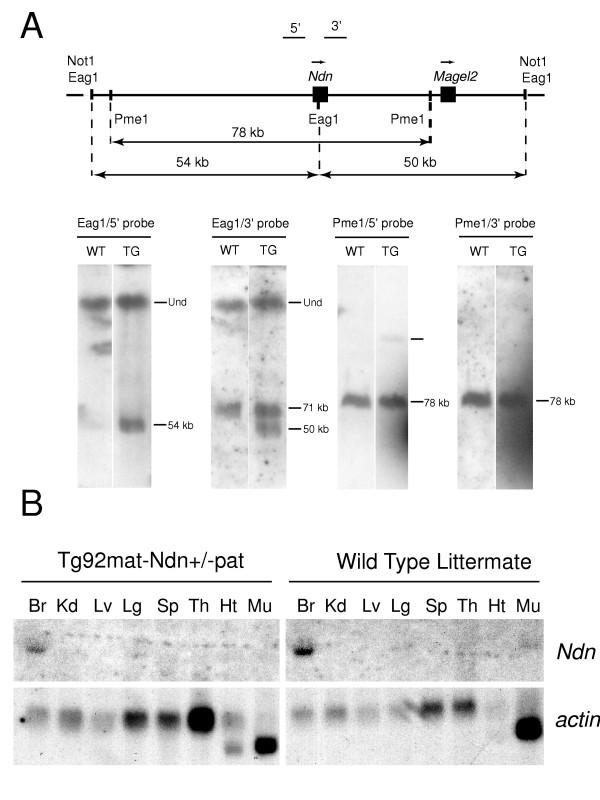
**Transgenic analyses. **(A) Structure and copy number of the BAC109 transgene in line Tg92. The BAC109 transgene, the relative positions of the *Ndn *and *Magel2 *genes, and the transgenic *Eag*I and *Pme*I restriction fragments detected with probes 5' and 3' are represented on the upper diagram. Genomic DNA was isolated from wild type (WT) or transgenic (TG) mice, digested by *Eag*I or *Pme*I, separated by PFGE, blotted and hybridized to the 5' or 3' probes. The presence of the 54 and 50 kb *Eag1 *transgenic fragments detected by the 5' and the 3' probes respectively demonstrate the integrity of the transgene. *Eag*1 is a methylation sensitive enzyme which explains the presence of a higher hybridization band corresponding to undigested methylated DNA (Und). Hybridization of *Pme*I digested genomic DNA with either the 5' or 3' *Ndn *probes, indicated that the BAC integrated in one full copy along with a truncated copy containing the whole 5' region upstream *Ndn *up to the *Ndn *promoter.(B) Non imprinted brain-specific expression of transgenic *Ndn*. *Ndn *expression was tested by Northern blot analysis. RNAs were isolated from Tg92mat-*Ndn*+/-pat or wild type littermate tissues: Br, brain, Kd, kidney, Lv, liver, Sp, spleen, Th, thymus, Ht, heart, Mu, muscles. Note that *Ndn *transgenic mRNAs are not detected in adult muscles whereas *Ndn *endogenous mRNAs are. After being hybridized to an intragenic *Ndn *probe, Northern blots were stripped and rehybridized to a β-actin probe to check for the presence and integrity of RNAs.

In order to determine if the spatio-temporal transcriptional regulation of PWS genes is dependant upon the IC, we initiated BAC transgenic analyses and chose to investigate the transcriptional regulation of the *Ndn *gene outside its natural genomic context. Since *Ndn *and *Magel2 *are co-regulated and the intergenic region between these two genes is around 30 kb only, we chose a bacterial artificial chromosome (BAC 109) containing both genes to generate transgenic mice (Fig. 4A). Three transgenic male founders were obtained and crossed with C57BL/6 females. Only one male transmitted the BAC109 transgene to its progeny and gave rise to the transgenic line 92 (Tg92) described in this study. The presence of BAC vector sequences allowed us to discriminate the transgenic from the wild type DNA. Genomic DNA from Tg92 mice was thoroughly analyzed by PFGE, Southern blot and PCR, to precisely determine the structure and the number of copy of the integrated transgene (Fig. 4A, data not shown). The transgene integrated in one full copy along with a truncated copy containing the whole 5' region upstream *Ndn *up to the *Ndn *promoter (Fig. 4A), near the centromeric region of chromosome 2, as determined by DNA FISH analysis (data not shown).

**Figure 5:**
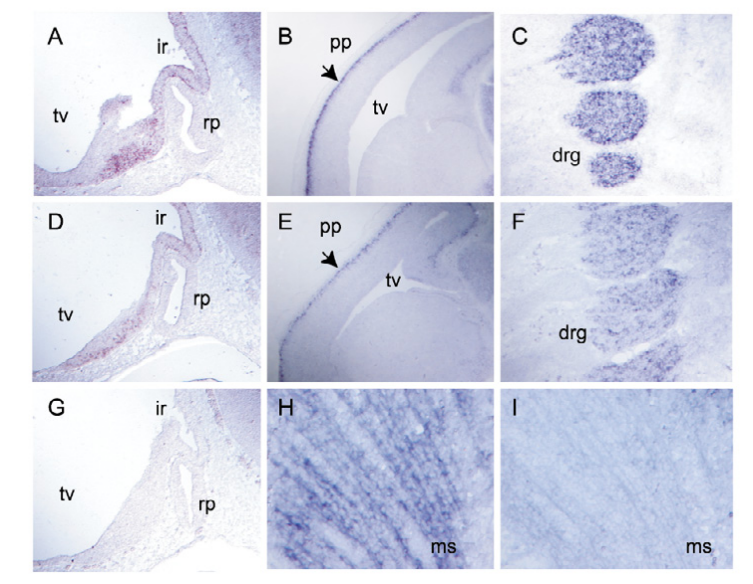
**Comparison of the wild type and transgenic *Ndn *expression profiles in E10.5 and E12.5 mouse embryos. ***In situ *hybridization was performed with an *Ndn *riboprobe on sagittal sections of wild type (A, B, C, H), Tg92mat-*Ndn*+/-pat (D, E, F, I) and Ndn+/-pat (G) embryos. The absence of *Ndn *signal in the E10.5 *Ndn*+/-pat embryo (G) demonstrates the specificity of the *Ndn *riboprobe. Similar expression profiles are shown in the infundibulum recess (ir) of E10.5 embryos (A, D), in the preplate of the telencephalic epithelium (pp) and in the dorsal root ganglia (drg) of E12.5 embryos. Note that no hybridization signal is detected in the transgenic Tg92mat-*Ndn*+/-pat E12.5 muscle (ms) (I) whereas endogenous *Ndn *is expressed in this tissue (H). rp, rathke pouch; tv, telencephalic vesicle.

Transgenic *Ndn *expression analysis was performed by Northern blot hybridization, on adult tissues of mice inheriting the transgene either maternally or paternally, on an *Ndn *null background (Tg92mat-*Ndn*+/-pat and Tg92pat-*Ndn*+/-pat, respectively). Whether maternally or paternally inherited, the transgenic *Ndn *gene was expressed with the same tissue specificity as the endogenous *Ndn *gene (Fig. 4B). Transgenic *Ndn *transcripts were only detected in the brain, although at a slightly lower level than endogenous transcripts. It should be noted that no transgenic *Ndn *gene expression was detected in muscle, a tissue in which endogenous *Ndn *transcripts are detected although at low levels as compared to the brain. *In situ *hybridization experiments were then performed on transgenic embryos carrying a paternal deletion of the *Ndn *allele (Tg92mat-Ndn+/-pat) and wild type mouse embryos, to compare the transgenic and endogenous profile of *Ndn *expression. Transgenic *Ndn *transcripts were detected in embryos from E10.5 as endogenous *Ndn *transcripts, and in the same structures, namely the central and peripheral nervous system (Fig. 5), at a slightly lower level in transgenic embryos as it was noted in transgenic adult tissues. No transgenic *Ndn *expression was detected in the dermo-myotome, the muscles or the tongue, although endogenous *Ndn *transcripts were detectable in these tissues (Fig. 5: H and I). Since *Magel2 *null mice were not available, *Magel2 *imprinting and expression from the BAC109 transgene could not be analyzed. However, no ectopic site of *Magel2 *expression was noted during embryogenesis.

Since the transgenic *Ndn *gene was not imprinted, methylation studies of the transgenic *Ndn *promoter region and CpG island were omitted.

### Search for *Ndn *transcriptional regulatory elements

**Figure 6:**
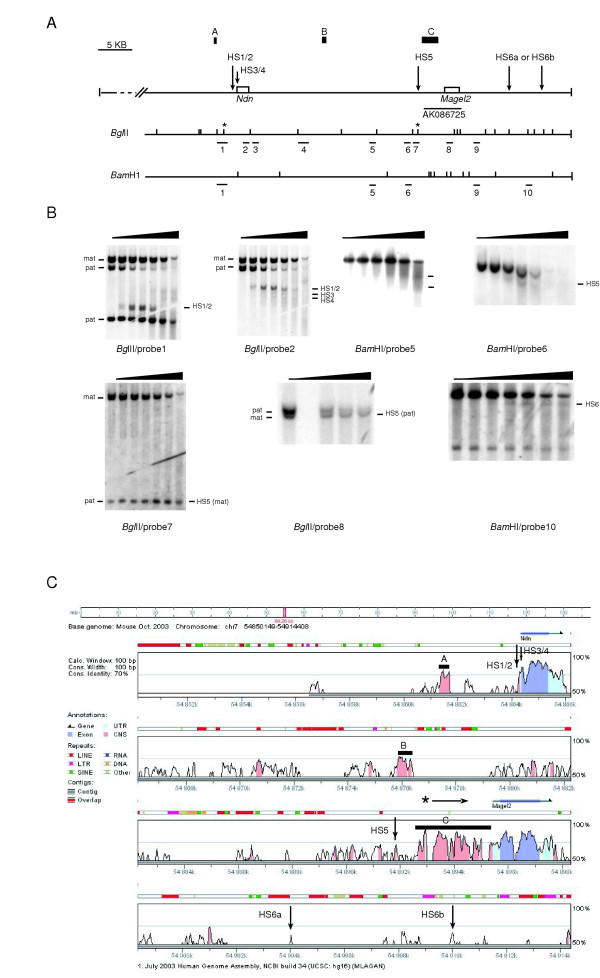
**Mapping of DNase I hypersensive sites in the *Ndn *and *Magel2 *genomic region. **(A) The diagram shows a map of the sequenced genomic region present in the BAC109. Localisation of HS sites is indicated by arrows in relation to the *Ndn *and *Magel2 *genes, and to the AK086725 EST. Position of *Bgl*II and *BamH*1 sites and probes 1 to 10 are indicated. Asterics indicate polymophic *Bgl*II restriction sites present on castaneus alleles detected with probes 1/2 and probes 7/8. A, B and C design three non-coding regions of homology between the mouse and the human genomes.(B) Southern blot analysis of *Bgl*II or *BamH*I cut genomic DNA isolated from brain nuclei treated with increasing concentrations of DNase I (from 0 to 100 U). Maternal (mat) and paternal (pat) alleles are indicated when discrimination is possible.(C) Sequence comparison between mouse and human *Ndn*/*Magel2 *genomic regions. Alignments were peformed with the genome VISTA program (window size 100 bp, homology threshold 70%). Conserved non-coding sequences (CNS) are depicted in pink, untranslated regions (UTR) and ORF in pale and dark blue respectively. Genes are indicated by blue arrows. Repeated elements are depicted above the sequences comparison. Mapped HS sites are indicated by vertical arrows. A, B and C design three non-coding regions of homology between the mouse and the human genomes. Although the C region is depicted as a non-gening sequence, recent isolation of ESTs corresponding to this region suggests that it might belong to the *Magel2 *transcription unit. The asterisk above the C region indicates the beginning of the ESTs.

Since *Ndn *was correctly expressed at least in the nervous system from the BAC transgene, *Ndn *regulatory elements must be present in the BAC109 genomic sequences. With the increasing availability of genomic sequence and the recent development of powerful global alignment algorithms, interspecies genomic sequence comparisons are becoming an efficient mean to identify conserved non-coding sequences (CNS) which regulatory potential can further be assessed experimentally [15,16]. However, since the whole human and mouse PWS domains are rather highly conserved which might render difficult to discriminate between functional and non functional CNS, we first undertook an experimental approach. We analyzed the chromatin DNase I sensitivity of the genomic sequences present in the BAC transgene. In the adult brain, hybridization of *Bgl*II- or *Bam*HI-cut DNA with a series of probes described in Fig. 1 led to the characterization of several major DNaseI hypersensitive sites associated to the *Ndn *gene (HS1/2, HS3 and 4) and of one site upstream the *Magel2 *gene (HS5) (Fig. 6). The probes used allowed a systematic analysis of the *Ndn*/*Magel2 *genomic sequences present in the BAC transgene, from -17.5 kb upstream the *Ndn *gene to17 kb downstream the *Magel2 *gene. Additional upstream *Ndn *sequences which were present in the BAC could not be analyzed since their sequences are not yet available.

Hybridization *of Bgl*II-cut DNA with probe 1 revealed two adjacent (~50–70 bp apart) and strong DNase I hypersensitive sites (HS1 and HS2) in the *Ndn *promoter region of the paternal allele (Fig. 6B). Hybridization with probe 2 revealed two additional but weaker DNase I hypersensitivity sites on the paternal allele (HS3 and HS4), localized in the *Ndn *5'UTR and at the beginning of the coding region, respectively. In contrast to the paternal allele, the maternal allele was highly resistant to DNase I digestion. In the adult kidney, a tissue in which *Ndn *is not expressed, the two parental alleles were highly and almost equally resistant to DNase I digestion (data not shown). The presence of DNase I hypersensitive sites HS1/2, HS3 and HS4 is therefore closely linked to the transcriptional activity of the *Ndn *gene since they were present on the paternal allele and only in *Ndn*-expressing tissues.

Hybridization of brain *BamH*1-cut DNA with probe 6 or *Bgl*II-cut DNA with probes 7 and 8 revealed one relatively strong DNase I hypersensitive site (HS5) 3.5 kb upstream the *Magel2 *gene, which was present on both parental alleles and co-localized with a polymorphic *Bgl*II site present on the CAST/Ei allele. HS5 was localized 1 kb upstream newly isolated full length *Magel2 *ESTs (AK086725, AK082944, AK086725; see Fig. 6A) which initiate around 2.5 kb 5' to the described *Magel2 *mRNA (NM013779) [17]. HS5 was present on both maternal and paternal alleles and in tissues in which *Magel2 *and/or *Ndn *are not expressed such as the adult kidney (data not shown) which suggests that this DNase I hypersensitive site is not linked to the transcriptional activity of these two genes.

No prominent DNase I hypersensitive site were detected in the *Ndn*-*Magel2 *intergenic region (excepted HS5). Multiple faint hybridization signals could however be detected with probes 3 and 4 on *Bgl*II-cut DNA (data not shown) and with probe 5 on *BamH*1-cut DNA, revealing regions of low DNase I hypersensitivity in the *Ndn*-*Magel2 *intergenic region (Fig. 6B). Finally, hybridization of probe 10 on brain *BamH*1-cut DNA allowed the detection of a lower hybridization band, revealing one or potentially two DNase I hypersensitive sites (HS6a or/and HS6b). We were not able to precisely localise the HS6 site(s) as well as its (their) parental origin.

We next mapped HS1 to 6 DNase I hypersensitive sites on a mouse/human sequence alignment (Fig. 6-C). The available mouse sequences used for this comparison start from -17.5 kb upstream the *Ndn *gene to the *Magel2 *extremity of BAC109. Several CNS defined as orthologous sequences greater than 100 bp and greater than 70% identity [15] were found and mainly gathered into three regions: upstream the *Ndn *gene (region A), in the intergenic *Ndn*-*Magel2 *region (region B) and in a 2.8 kb upstream the *Magel2 *gene (region C). None of the hypersensitive sites identified in this study mapped to region A, B or C. HS1/2 which are closely linked to the transcriptional activity of the *Ndn *gene are localized in a CNS immediately upstream the *Ndn *gene. HS5 co-localized to an isolated CNS localized 0.7 kb upstream the highly conserved C region and around 1 kb upstream the newly described full length *Magel2 *ESTs. However, the functional significance of HS5 in the *Magel2 *and/or *Ndn *gene transcriptional regulation remains to be determined since this site was neither allele nor tissue-specific. It should be noted that there was almost no conservation of sequence in the region downstream the *Magel2 *gene. In contrast, the intergenic *Ndn*-*Magel2 *region was relatively well conserved and regions of low DNase I hypersensitivity were detected in this region which might have functional importance.

## Discussion

### Coordinated central nervous system-specific expression of PWS genes

Study and comparison of *Snurf*-*Snrpn*, *MBII*-*85*/-*52 *snoRNAs, *Ndn *and *Magel2 *expression profiles brought new information on the transcriptional regulation of these genes. *Snurf*-*Snrpn*, the snoRNAs *MBII*-*52 *and *MBII*-*85 *and the distantly located *Ndn *and *Magel2 *genes were expressed in the mouse developing nervous system at E10.5 and E13.5 with strikingly similar patterns in the central nervous system.

More divergent expression patterns were observed in the peripheral nervous system and non-neuronal tissues.

Accordingly, *Snurf*-*Snrpn*, *MBII*-*85 *and *MBII*-*52 *snoRNAs were exclusively expressed in the developing nervous system, and in identical structures in the central nervous system. Marked differences of expression between *Snurf*-*Snrpn *and the snoRNAs were found in cranial and dorsal root ganglia, structures in which *Snurf*-*Snrpn *was highly expressed whereas the snoRNAs were expressed at very low levels (*MBII*-*85*) or not at all (*MBII*-*52*). These results reinforce the proposal that the snoRNAS derive from the processing of long primary transcripts initiated at the U exons, upstream *Snurf*-*Snrpn *[18] (Le Meur et al., submitted). It is interesting to note that *Snurf*-*Snrpn *was previously reported to be ubiquitously expressed in adult tissues [12], whereas we found it to be exclusively expressed in the nervous system at E10.5 and 13.5 of mouse embryogenesis.

As previously reported [14,11], *Ndn *and *Magel2 *mRNAs were detected almost exclusively in the nervous system of the mouse embryo from E9.5/10.5 onwards, and although *Magel2 *transcripts were detected at much lower levels than *Ndn *transcripts, these two genes displayed almost identical expression patterns in the central nervous system (brain and spinal chord). Particularly striking was their identical pattern of expression in the E10.5 embryo. At later developmental stages, *Magel2 *expression domain became more restricted but remained included in *Ndn *expression domain. *Magel2 *transcripts were however detected at very low levels in almost all the structures in which *Ndn *was expressed, including non neuronal structures such as the mandibular component of the first branchial arch, the tongue and the muscles. *Magel2 *transcripts have been reported to be particularly instable due to the presence of AU-rich elements (ARE) in their 3' untranslated region [17] and this could explain why they were detected at such low levels.

Our expression analysis in the mouse embryo indeed suggests the existence of a central nervous system-specific neural regulatory element coordinating the PWS genes expression. *Ndn *and *Magel2*, specifically, could share additional regulatory elements.

### Co-expression of PWS genes and the IC

We further emitted the hypothesis that the central nervous system-specific regulatory element could be physically associated to the PWS IC because of the proposed evolutionary history of the PWS domain and because the IC is involved in coordinating long range chromatin modifications leading to imprinted expression of genes in the whole domain. The *Snurf*-*Snrpn *locus with its associated IC has been proposed to be the most ancestral transcriptional unit of the domain [12,13]. Other PWS genes (*Ndn*, *Magel2*, *Mkrn3 *and *Frat3*) would have later been acquired by retroposition and/or local *cis*-duplication of retroposed genes and would have adopted the same transcriptional regulation as the *Snurf*-*Snrpn *transcriptional unit. Unless retroposons arise from reverse-transcriptase-mediated processing of aberrant or alternative transcripts including endogenous promoter elements, their functionality is dependent upon regulatory elements present at their site of insertion. Retroposons in the PWS region could have therefore adopted the *Snurf*-*Snrpn *regulation both for imprinted and spatio-temporal expression. Finally, the fact that paternal deletions of the IC abolish the expression of all the PWS genes [8,7] also suggests a possible intertwining between regulatory elements controlling both imprinting and spatio-temporal expression.

Our study did not however confirm that the IC might coordinate the spatio-temporal expression of PWS genes. Our transgenic analysis clearly showed that *Ndn *does not rely on the IC for its spatio-temporal expression. *Ndn *was correctly regulated from the BAC transgene lacking the IC both in the developing embryo and in adult tissues. These results demonstrate that the *cis*-regulatory sequences involved in both the developmental and tissue-specific expression of *Ndn *were present in the transgene and were not associated to the IC. The fact that the IC was not involved in the spatio-temporal regulation of the *Ndn *gene does not however exclude its putative involvement in the spatio-temporal regulation of the *Snurf*-*Snrpn *transcriptional unit. No transcriptional regulatory elements or sequences excepted those involved in the imprinted expression [19] have yet been characterized in the *Snurf*-*Snrpn *transcriptional unit.

### Search for *Ndn *and *Magel2 *regulatory elements

Since the *cis*-regulatory sequences involved in both the developmental and tissue-specific expression of *Ndn *were present in the transgene and since we showed that *Ndn *and *Magel2 *expression was coordinated both in the central nervous system and in some non neuronal tissues of the mouse embryo, we searched for *Ndn *and *Magel2 *regulatory elements in the BAC109 genomic sequences. *Ndn *and *Magel2 *are two evolutionary related retrotransposons, which belong to the MAGE D gene family [20]. Phylogenetic studies could not predict whether these two genes arose from two distinct retroposition events or whether they arose from the initial retroposition of one of these two genes followed by a *cis*-duplication event (Blanc M., unpublished observations). Their close genomic localisation, within 30 kb in the mouse genome, would however be in favour of this second hypothesis. The co-expression of *Ndn *and *Magel2 *could therefore result from regulatory elements sharing and/or duplication. Intraspecies genomic comparisons of mouse or human *Ndn *and *Magel2 *upstream sequences did not reveal any significant homology which does not exclude the possibility that one of these two genes arose by a *cis*- duplication. Our experimental search for regulatory elements elements in the *Ndn*/*Magel2 *genomic sequences was limited to sequences available in the databases and was therefore not exhaustive. However, we found several DNase I hypersensitive sites (HS1/2, HS3 and 4) linked to *Ndn *transcriptional activity and associated to the *Ndn *promoter. Previous transgenic studies in the zebrafish using a series of hybrid transgenes containing various mouse *Ndn *promoter sequences associated to the reporter LacZ gene suggested that the mouse *Ndn *promoter from -845 bp to +63 bp functioned in the zebrafish embryo in a temporal, spatial and tissue-specific manner [21]. In particular, a *cis*-acting element driving the neuronal-specific expression was located into an 87 bp sequence from -173 to -87 bp of the *Ndn *gene which exactly co-localizes with HS1/2. No identified transcriptional factor brain-specific binding site could however be identified in this sequence. Deletion of promoter sequences in *Ndn*-KO mutant mice [22] did not affect *Magel2 *transcriptional regulation (Watrin F., data not shown) which suggests that if the coordinated *Magel2 *and *Ndn *expression results from the sharing of a putative enhancer, this enhancer is not associated to *Ndn *promoter sequences.

The human *NDN *gene has been shown to be expressed in a larger panel of tissues than the mouse *Ndn *gene [23,24] but our data suggest that these two genes might have similar expression profiles in the nervous system [23] (data not shown). An interspecific (mouse/human) comparison of available genomic sequences was therefore performed in order to identify genomic sequences involved in central nervous system expression. This comparison did not allow the identification of such sequences but nevertheless brought some new information on the *Magel2 *transcriptional unit, showing the existence of a 2.5 kb highly conserved region upstream the described *Magel2 *gene. Identification of full length embryonic and brain specific *Magel2 *ESTs including this region further confirms that this region belong to the *Magel2 *transcriptional unit.

In view of our results, expression of the *Ndn *and *Magel2 *genes could be coordinated by a common enhancer distinct from *Ndn *promoter sequences and which could be localized in sequences upstream the *Ndn *gene that we could not analyze. It should be noted that the *Ndn*/*Magel2 *intergenic region was rather well conserved and harbored several regions of low DNase I hypersensitivity which might deserve further investigation.

### The transgene is not imprinted

As expected, our transgene which was physically separated from the IC was not imprinted. When outside of its natural genomic context, *Ndn *was expressed from either parental allele. Whether particular genomic sequences around or associated to *Ndn *such as the 5' CpG island are necessary to respond to the primary imprinting signal established at the IC remains to be determined. An imprinted brain-specific *Ndn *non coding (nc) antisense RNA (PX00010K13; DDBJ accession no. AK14392) initiated in (or including) the 5' part of *Ndn *coding sequence and extending in *Ndn *upstream sequences has recently been described and according to the authors, this antisense transcript could be involved in the imprinted expression of *Ndn *mRNA [25]. This transcript could potentially be transcribed from our transgene. We did not detect this antisense nc transcript in transgenic brain RNA which is coherent with the fact that the transgene was not imprinted. However, we did not detect it in wild type brain either (data not shown). In one of the mouse models in which part of the *Ndn *CDS -and therefore the region in which the antisense transcript is initiated- was deleted [26], the *LacZ *gene which replaced the *Ndn *was monoallelically expressed, further strengthening an absence of role of this hypothetical antisense transcript in *Ndn *imprinting.

## Conclusions

Our analysis strongly suggests the existence of a central nervous system-specific regulatory element which would coordinate expression of the PWS genes in the developing mouse embryo and we proposed that this element could be associated to the IC. However, our transgenic studies clearly demonstrate that when physically separated from the IC, a transgenic *Ndn *gene, although not imprinted anymore, can still be correctly expressed in the developing nervous system of the mouse embryo. The BAC transgene therefore contained the regulatory elements needed for the spatio-temporal expression of *Ndn *(and most likely *Magel2*) in the developing nervous system. Three hypotheses can be made: 1) the PWS genes co-expression that we observed is incidental, 2) the element(s) localised in genomic sequences surrounding the *Ndn*/*Magel2 *and which regulate(s) *Ndn *(and *Magel2*) expression could also regulate other PWS genes, 3) several central nervous system-specific regulatory elements resulting from duplication events might be present in the PWS domain. The evolutionary history of the PWS domain is more in favour of the third hypothesis but will need further investigations to be confirmed.

## Methods

### Mice

Adult C57BL/6 and C57BL/6 X CBA mice were purchased from IFFA CREDO, and *M. musculus castaneus *(CAST/Ei) male mice from the Jackson Laboratory. Mice carrying an *Ndn *null mutation on a 129/Sv genetic background [22] and transgenic Tg92 mice were bred in-house.

### *In situ *hybridization

All *in situ *hybridization experiments for the study of PWS genes expression were performed on C57BL/6 embryos. *In situ *hybridization was performed on 14 μM paraformaldehyde-fixed cryosections with antisens digoxigenin-labeled riboprobes. Sections were washed in 1X PBS, treated with RIPA buffer (150 mM NaCl, 1% NP-40, 0.5% Na deoxycholate, 0.1% SDS, 1 mM EDTA, 50 mM Tris pH 8), post-fixed in paraformaldehyde, acetylated and further washed in PBST before the hybridization step. All hybridization and post-hybridization washes were performed at 70°C. Hybridization was performed in 50% formamide, 5X SSC, 5X Denhart's solution (Sigma), 0.5 mg/ml herring sperm DNA, 0.25 mg/ml yeast RNA. Washes were performed in 50% formamide, 2X SSC, 0.1% Tween 20. Digoxigenin labelling was detected using anti-digoxigenin Fabs (Roche Biochemicals) coupled to alkaline phosphatase and NBT/BCIP (Sigma). The *Ndn *riboprobe (290 bp) hybridizes to the 3' UTR of the *Ndn *mRNA (nt 2130 to 2420; accession number D76440). The *Magel2 *riboprobe (318 bp) hybridizes to the 3' part of the *Magel2 *ORF (nt 3730 to 4048; accession number AK086725). The snoRNAs *MBII*-*85 *and *MBII*-*52 *riboprobes were synthesized from plasmids given by J. Cavaillé. The *Snurf *riboprobe (277 bp) hybridizes to *U*/*Snurf*-*Snrpn *transcripts and recognizes sequences from the 3' end of the *Snurf *exon 1 to the 3' end of the *Snurf *exon 3 (nt 3639 to nt 77687; accession number AF332579).

### Generation and breeding of transgenic mice

BAC109 was isolated by hybridization of mouse bacterial artificial (BAC) high-density membranes (Research Genetics, Inc, USA) with a genomic *Ndn *probe, and contains a 103 kb *Not*I insert comprising the *Ndn *and *Magel2 *genes. The *Ndn *gene is localized in the center of BAC109, the *Eag*I intragenic site being situated 54 and 50 kb away from the vector *Not*I sites and the 3' extremity of the *Magel2 *gene is localized 17 kb from one of the vector *Not*I site (Fig. 3). Unlinearized cesium chloride gradient purified BAC109 DNA was resuspended in injection buffer (10 mM Tris-HCl pH 7.5, 0.1 mM EDTA pH 8.0, 100 mM NaCl, 30 μM spermine, 70 μM spermidine) at 3 ng/μl and microinjected in the pronucleus of C57BL/6 X CBA fertilized eggs. Founders were identified by Southern blot analysis using a PCR probe amplified from the pBeLoBAC11 vector and a *Bgl*II digestion, and subsequently bred on a C57BL/6 genetic background. Transgene copy number and integrity were determined by PFGE and Southern blot analysis. The 5' and 3' probes used in the Southern blot analysis correspond to the probes 1 and 2 described in the "DNase I Hypersensitivity Mapping" section. Transgenic *Ndn *expression analysis (Northern blot and *in situ *hybridization) was performed on embryos or adult mice inheriting the transgene either paternally or maternally, on a *Ndn *null background. These embryos or adult mice were obtained by mating transgenic males carrying the *Ndn *null mutation to wild type C57BL/6 females (Tg92pat-*Ndn*+/-pat) or transgenic females to males carrying the *Ndn *null mutation (TgBAC92mat-*Ndn*+/-pat).

### DNase I hypersensitivity mapping

Tissues were dissected either from inter-specific F1 hybrids between *M. musculus *C57BL/6 females and *M. musculus castaneus *males (B6 x CAST/Ei) F1 or from inbred C56BL/6 mice (B6). Nuclei were isolated from adult mouse tissues as described [27]. Nuclei were resuspended in DNase I digestion buffer (0.3 M sucrose, 60 mM KCl, 15 mM NaCl, 5 mM MgCl_2_, 0.1 mM EGTA, 15 mM Tris-HCl pH 7.5, 1 mM DTT, 0.2 mM PMSF, 0.4 mM CaCl_2_, 5% glycerol) at a concentration of 5.10^7 ^nuclei/ml. Increasing amounts of DNase I (bovine pancreas grade I; Boehringer Mannheim) from 0 to 100 units diluted in 5 μl of DNase I digestion buffer were added to 95 μl aliquots of nuclei. After a 1 min incubation at 25°C, the digestion was stopped by addition 100 μl of stop solution (20 mM EDTA, 1% SDS, 0.1 mg/ml proteinase K) and samples were incubated overnight at 37°C. Genomic DNA was purified by multiple phenol/chloroform extractions and precipitated with ethanol. After resuspension, the DNA was digested with the appropriate restriction enzymes, separeted by gel electrophoresis, and transferred to Hybond N+ membranes. The membranes were hybridized by random hexamer- radiolabeled probes, in church solution (0.5 M NaPi pH 5.5, 7% SDS), at 65°C. Filters were washed three times in 0.2X SSC, 0.1% SDS at 65°C and exposed to X-ray film at -70°C. When necessary, blots were stripped by incubation in 0.1X SSC, O.1% SDS at 95°C. Most probes were prepared by PCR amplification of BAC109 sequences, using the following primers: Probe 1: S-5'-AGATCTGAAGACATAATG-3', AS-5'-GCTCTCCATTTCTAT TAGGTC-3'; Probe 2:S-5'-ATAAGTATTTGGTACTTTCAC-3', AS-5'-TGCTAAGTGCCTACACTGAG3'; Probe 3: S-5'-GAGCGAAACTATTCTGACAG3', AS-5'-AAGCTTCCTCCTCTATGGCAA-3'; Probe 4: S-5'-GATTTCTGCTAAGATTGG-3', AS-5'-ATGTTCCCTCTAGAAACC-3'; Probe 6: S-5'-AGTTAGAGACAAGCCTAG-3', AS-5'-TTCTGGGATGTCTCAGGA-3'; Probe 7: S-5'-GCATTTTGAGGAAGTACCCA-3', AS-5'-CATGGCCATTTCTAACTGTG-3'; Probe 8: S-5'-CCAAGGAGCTTGGAGGGC-3', AS-5'-CTCGTAGAGTGCGGCCAA-3'; probe 9: S-5'-ACATCAATAGTTTGATAC-3', AS-5'-GGGTGTGGCTGTGCATTGTT-3'

## Authors' contributions

FW drafted the manuscript, designed the study, and carried out the PWS genes expression analysis, the transgenic analysis and the search for transcriptional regulatory elements. ELM contributed to the PWS gene expression analysis. NR carried out molecular studies on the transgenic lines and participated to the search for DNase I hypersensitive sites. LD was involved in the design of the transgenic studies. MR injected the transgene and established the transgenic lines. FM coordinated the project and participated to the manuscript draft.
